# Confirmatory Factor Analysis of the Perceived Stress Scale-10 Across Age Groups

**DOI:** 10.1093/geroni/igaa057.975

**Published:** 2020-12-16

**Authors:** Robert Intrieri, Paige Goodwin

**Affiliations:** Western Illinois University, MACOMB, Illinois, United States

## Abstract

The Perceived Stress Scale-10 (PSS; Cohen et al, 1983) was developed to measure subjective elements of stress. Most measures focus on objective characteristics of stress (e. g., frequency of occurrence) and specific situations that produce stress (e. g., divorce) but ignore the cognitive appraisal associated with stressful stimuli. The PSS-10 assesses the interplay between stressor and appraisal-mediated coping ability. Factor analytic studies provide support for two factors: perceived helplessness and perceived self-efficacy (see Roberti et al. 2006). The current study presents data from 591 people across three groups: 221 young adults (mean age 19.31, SD = 1.21), 283 middle-age adults (mean age 48.27, SD = 5.14), and 109 older adults (mean age 72.95, SD = 7.22). An ordinal confirmatory factor analysis (CFA) using robust weighted least squares (WLSMV) evaluated invariance across age groups. Results showed CFI/TLI values of .964/.953, 965/.960, and .964/.969 for Configural (CI), Metric (MI), and Scalar (SI) models. The RMSEA for CI, MI, and SI models was .086, .081, and .071. Based upon recommendations of Cheung and Rensvold (2002), Sass (2011), and Chen (2007), a cutoff of ΔCFI ≥ 0.01 was established as evidence of invariance. The ΔCFI between CI and MI models was < .01 so analysis continued with the SI test. Once again, ΔCFI between MI and SI models was < 0.01 which did not justify rejection of the null hypothesis. Based on these analyses, PSS-10 scores are valid across multiple age groups. Further, results support the multidimensional nature of the PSS-10.

